# NanoCoV19: An analytical pipeline for rapid detection of severe acute respiratory syndrome coronavirus 2

**DOI:** 10.3389/fgene.2022.1008792

**Published:** 2022-09-15

**Authors:** Jidong Lang

**Affiliations:** Department of Bioinformatics, Qitan Technology (Beijing) Co., Ltd., Beijing, China

**Keywords:** nanopore sequencing technology, SARS-CoV-2, hotspot mutation, phylogenetic tree, coronavirus disease 2019 (COVID-19)

## Abstract

Nanopore sequencing technology (NST) has become a rapid and cost-effective method for the diagnosis and epidemiological surveillance of severe acute respiratory syndrome coronavirus 2 (SARS-CoV-2) during the coronavirus disease 2019 (COVID-19) pandemic. Compared with short-read sequencing platforms (e.g., Illumina’s), nanopore long-read sequencing platforms effectively shorten the time required to complete the detection process. However, due to the principles and data characteristics of NST, the accuracy of sequencing data has been reduced, thereby limiting monitoring and lineage analysis of SARS-CoV-2. In this study, we developed an analytical pipeline for SARS-CoV-2 rapid detection and lineage identification that integrates phylogenetic-tree and hotspot mutation analysis, which we have named NanoCoV19. This method not only can distinguish and trace the lineages contained in the alpha, beta, delta, gamma, lambda, and omicron variants of SARS-CoV-2 but is also rapid and efficient, completing overall analysis within 1 h. We hope that NanoCoV19 can be used as an auxiliary tool for rapid subtyping and lineage analysis of SARS-CoV-2 and, more importantly, that it can promote further applications of NST in public-health and -safety plans similar to those formulated to address the COVID-19 outbreak.

## 1 Introduction

Severe acute respiratory syndrome coronavirus 2 (SARS-CoV-2), a causative agent of coronavirus disease 2019 (COVID-19), was identified in late 2019 (Zhu et al., 2020). Shortly thereafter, SARS-CoV-2 spread around the world, causing significant social problems, medical-system stress, and economic stagnation in all countries. It is a positive-sense single-stranded RNA virus with a 29,903 bp genome size, which was published in full in January 2020 (Lu et al., 2020a; Wu et al., 2020). Such publication led to the development of assays for SARS-CoV-2 detection based on real-time polymerase chain reaction (RT-PCR), which has been commonly used as a gold standard for monitoring the COVID-19 pandemic (van Kasteren et al., 2020). Sequencing the genomes of SARS-CoV-2 at different times and locations and in different populations yields information related to the viral-mutation rate, transmission dynamics, and origin of the disease (Boni et al., 2020). It is also a key technique for understanding the viral lineages that circulate in individual countries and understanding how frequently new variant sources from other geographic regions are introduced. Genome sequencing of SARS-CoV-2 therefore serves to indicate the success of control measures, allow an understanding of how the virus evolves in response to interventions, and inform public response by defining the phylogenetic structure of the disease’s outbreaks (Rambaut et al., 2020). Integration of the complete viral genomes and detailed epidemiological data provides a valuable reference for epidemiological investigations into transmission networks and inferences of where cases of unknown origin might have arisen (Lu et al., 2020b; Fauver et al., 2020; Gonzalez-Reiche et al., 2020; Gudbjartsson et al., 2020; Rockett et al., 2020). In addition, several studies have shown that different lineages of SARS-CoV-2 can infect the same person (Fonseca et al., 2021; Tillett et al., 2021; To et al., 2021). Sequencing and analysis of the SARS-CoV-2 genome are essential to confirm reinfections and to rule out disease recurrence. Rapid and reliable sample sequencing in environments such as hospitals is essential to such epidemiological surveillance. Furthermore, large-scale longitudinal monitoring of SARS-CoV-2 genomes also provides important information on the virus’s evolution, with important implications for COVID-19 vaccine development (Korber et al., 2020; Li et al., 2020; Uddin et al., 2020; Young et al., 2020).

Excitingly, nanopore sequencing technology (NST) has demonstrated its feasibility and effectiveness in epidemiological surveillance during outbreaks of viral diseases such as Ebola and Zika (Quick et al., 2015; Quick et al., 2016; Quick et al., 2017). Some studies have developed several methods of rapidly sequencing SARS-CoV-2 genomes based on nanopore sequencing platform of companies represented by Oxford Nanopore Technologies (ONT), which is critical for rapid diagnosis and monitoring of the spread of the new coronavirus (Bull et al., 2020; Wang et al., 2021a; Jia et al., 2021). However, the principles and data characteristics of NST, such as non-random systemic errors and many unexpected indels, have a certain effect on analytical results (Magi et al., 2017; Bull et al., 2020). In addition, due to the timeliness requirements of the turnaround time, the sequencing platforms used for SARS-CoV-2 are still primarily based on next-generation sequencing (NGS), with analytical methods mainly focused on the presence of targeted gene regions on the genome. Therefore, we developed an analytical pipeline for rapid detection and lineage identification of SARS-CoV-2, named NanoCoV19, based on NST combined with phylogenetic-tree and hotspot mutation analysis, to distinguish the new coronaviral lineages. We hope that NanoCov19 can further the application of NST in monitoring the direction of COVID-19 outbreaks.

## 2 Materials and methods

### 2.1 NanoCoV19 analytical principle

NanoCoV19 consists of two parts: the construction of a reference database, and the data analysis pipeline.

#### 2.1.1 Construction of reference genome sequence and mutation hotspot database for analysis

**FIGURE 1:**
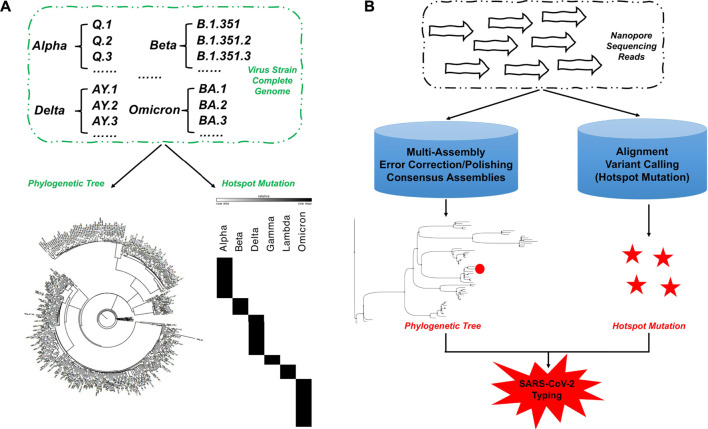
Schematic diagram showing the analytical principle of NanoCoV19. **(A)** Construction of a reference sequences and hotspot mutations database. **(B)** Pipeline for lineage analysis of SARS-CoV-2 based on nanopore sequencing data.

We downloaded the lineage information of the alpha, beta, gamma, delta, lambda, and omicron variants from RCoV19 (version 4.0) and the corresponding complete genome sequence of SARS-CoV-2 from the National Center for Biotechnology Information (NCBI; Bethesda, MD, United States) virus database (The date of data release used for this paper was 1 June 2022). One genome sequence was randomly selected from the lineage of each variant as a representative reference sequence database (Supplementary Table S1) for phylogenetic-tree analysis. We used MAFFT (v7.487) (Katoh et al., 2002) to perform multiple-sequence alignment on these sequences, and iqtree2 (v2.1.4-beta) (Nguyen et al., 2015) to perform phylogenetic-tree analysis. FigTree (v1.4.4) (https://github.com/rambaut/figtree) was used for visualization to determine whether the selected reference sequences discriminated between viral lineages (Figure 1A).

We also randomly selected 10 complete genome sequences from each lineage. For lineages with < 10 complete genome sequences, all sequences were included in the group. Then, we used NanoSim-H (v1.1.0.4) (Yang et al., 2017) to simulate the error-free nanopore sequencing data of *n* × 1000 sequencing reads, where *n* represents the number of complete genomes contained in each variant (Supplementary Table S2). The reference genome (MN908947.3) of SARS-CoV-2 was downloaded from the NCBI database. We used Minimap2 (v2.21-r1071) (Li, 2018) to do the read alignment, and followed by Sambamba (v0.8.0) (Tarasov et al., 2015) for alignment file processing. Longshot (v0.4.1) (Edge and Bansal, 2019) was used to detect mutations. Finally, we used mutation results that were unique to each variant and also present in the *lineages.csv* information published on RCoV19 as a database of hotspot mutations for distinguishing lineages (Figure 1A; Supplementary Table S3).

#### 2.1.2 Data analysis pipeline

As shown in Figure 1B, raw nanopore sequencing data was pre-processed using Porechop (v0.2.4; https://github.com/rrwick/Porechop). Next, we performed statistical analysis on the preprocessed clean data using NanoPlot (v1.38.0) (De Coster et al., 2018), after which we employed FlyE (v2.8.3-b1695) (Kolmogorov et al., 2019), Raven (v1.8.1) (Vaser and Šikić, 2021), Canu (Koren et al., 2017), Wtdbg2 [v0.0 (19830203)] (Ruan and Li, 2020), and Trycycler (v0.5.3) (Wick et al., 2021) for data assembly and generation of consensus sequences. Racon (v1.4.20) (Vaser et al., 2017) was used for correction and self-correction after each assembly. In the presence of NGS sequencing data, we polished each error-corrected assembly sequence using Pilon (v1.24) (Walker et al., 2014). We used Samtools (v1.12) (Li et al., 2009) to process the alignment files, and soap.coverage (v2.7.7; https://github.com/gigascience/bgi-soap2/tree/master/tools/soap.coverage) was used for statistical analysis of sequencing depth and genome coverage. The software and parameters used for establishing phylogenetic-tree and hotspot mutation detection were consistent with those described in part (Zhu et al., 2020).

### 2.2 Testing data set

Ten complete genome sequences that differed from the constructed reference database were randomly selected from the complete genomes of the alpha, beta, gamma, delta, lambda, and omicron variants as data for testing the analytical pipeline. We used NanoSim-H (v1.1.0.4) to simulated nanopore sequencing reads with and without errors. The number of simulated reads was 1000 (Supplementary Table S4). We used nucmer (v3.1; *−mum*) (Marcais et al., 2018) to compare and analyze the assembled draft genome and the corresponding complete genome.

To evaluate the real-world performance of NanoCoV19, the nanopore sequencing data published by Afrad et al. (2021) were also downloaded.

## 3 Results

### 3.1 NanoCoV19 performed well on the testing data set

**FIGURE 2:**
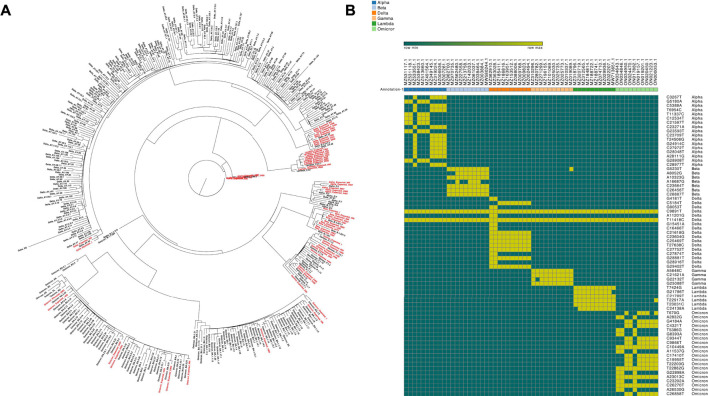
Analytical results of simulated sequence data for 60 lineages. **(A)** The result of phylogenetic tree analysis (the red text represents simulated data). **(B)** The heatmap analysis of hotspot mutations.

We directly analyzed the phylogenetic tree and detected hotspot mutations of 10 randomly selected complete genomes of the six SARS-CoV-2 variants. The results of phylogenetic-tree (Figure 2A) and hotspot mutation (Figure 2B) analysis were consistent with our expectations: i.e., the concordance rate was 100%. Further analysis of the 15 SARS-CoV-2 sub-lineage B.1.617.2 strains published by Afrad et al. (2021) showed that the predicted hotspot mutations were all delta variants (Supplementary Table S5), which was consistent with the classification of pangolin lineage B.1.617.2. However, because the read lengths of the sequencing data were all < 1,000 bp, which was the minimum overlap required, FlyE did not generate effective assembly results, making it impossible to carry out more-detailed lineage analysis.

### 3.2 The accuracy and integrity of assembly affected the phylogenetic-tree analysis

**FIGURE 3:**
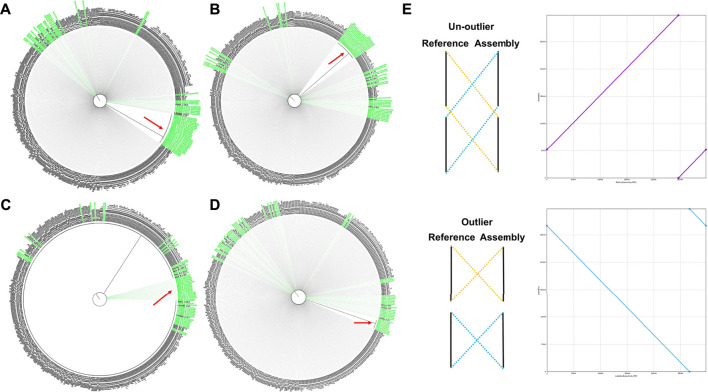
Assembly accuracy affects phylogenetic tree analysis. **(A)** The assembly results of FlyE to analyze simulated data with errors. **(B)** The assembly results of FlyE to analyze simulated data without errors. **(C)** The assemblies and consensus results of Trycycler to analyze simulated data with errors, which combination with 10 high-quality assembly results. **(D)** The assemblies and consensus results of Trycycler to analyze simulated data with errors, which combination with 23 high-quality assembly results. **(E)** The structural problems of the assembled draft genomes resulted in the outlier samples, which could not effectively distinguish the lineage.

We used only FlyE assembly results to analyze simulated read data with and without errors. Our results showed that our hotspot mutation analysis results were accurately and effectively for lineage subtyping (Supplementary Tables S6, S7). However, 28 (Figure 3A) and 21 (Figure 3B) simulated samples with and without errors, respectively, were not effectively distinguished after assembly but formed a unique branch and were defined as outlier samples. The remaining assembly results were accurately and effectively performed lineage subtyping. By comparing the assembly results of the outlier samples with their corresponding complete genomes, we found that the outlier results might have been due to the structural problems of the assembled genomes (Figure 3E), indicating that the requirements for completeness and accuracy of the assembly results would be very high when performing cluster analysis on phylogenetic trees. Maybe too many indels or sequence structure problems would lead to serious errors and even failure of lineage analysis, which also reflecting the necessity of comprehensive analysis combined with hotspot mutation analysis.

For the simulated data with errors, we used the assembly results of Raven, FlyE, and Wtdbg2 to combine 10 high-quality assembly results (i.e., the complete genome sequences published by the corresponding lineages). Trycycler was also used to generate consensus sequences. This significantly improved the results: the number of outlier samples dropped to 18 (Figure 3C). Subsequently, after we added 23 high-quality assembly results (the maximum number of sequences that could be input into Trycycler is 26), the number of outlier samples was only 8 (Figure 3D). The lineage analysis results of the remaining simulated data were basically correct.

### 3.3 Overall analysis time could be controlled within 1 h

Analysis of the 1000-read data from five testing samples showed that on an AMD EPYC 7542 32-core processor with 2 T of memory and 128 processors, when we used 16 processors for each task, the overall analysis time of NanoCoV19 analytical pipeline was controlled within 1 h (Table 1).

**TABLE 1:** Running time during each step of the five tests.

Testing sample		Alpha	Beta	Gamma	Lambda	Omicron
Compute resource	AMD EPYC 7542 32-core processor, 2T memory, 128 processor (16 processor/task)					
Data size	Read number	1,000	1,000	1,000	1,000	1,000
	Base number	7,759,122	7,869,879	7,784,216	7,485,683	7,638,683
	Read length N50	9,496	9,553	9,469	9,168	9,134
Data analysis	Data preprocessing	0:05:40	0:06:20	0:05:01	0:07:12	0:05:07
	Assembly-FlyE	0:01:57	0:02:01	0:02:01	0:01:57	0:01:56
	Assembly-Canu	0:02:08	0:02:07	0:02:06	0:01:58	0:02:01
	Assembly-Wtdbg2	0:00:06	0:00:13	0:00:07	0:00:05	0:00:11
	Assembly-raven	0:00:03	0:00:02	0:00:03	0:00:02	0:00:03
	Racon	0:00:15	0:00:21	0:00:21	0:00:18	0:00:18
	Pilon	0:11:16	0:10:44	0:10:28	0:09:52	0:09:08
	Trycycler	0:00:38	0:00:38	0:00:43	0:00:41	0:00:40
	Phylogenetic tree	0:33:58	0:35:27	0:23:48	0:22:28	0:23:02
	Variation calling	0:00:07	0:00:06	0:00:11	0:00:06	0:00:07
	Total time	0:56:08	0:57:59	0:44:49	0:44:39	0:42:33

## 4 Discussion

The development of NST has been very rapid (Magi et al., 2018; Wang et al., 2021b), and exciting results have been achieved in many fields, especially metagenomics for pathogen detection (Charalampous et al., 2019; Gu et al., 2021) and animal and/or plant genome assembly (Loman et al., 2015; Vaser et al., 2017; Lang et al., 2022a). Importantly, the advantages of NST in real-time sequencing analysis are self-evident (Payne et al., 2021; Goenka et al., 2022). NST has played a critical role in the tracing and rapid detection of outbreaks of infectious diseases such as COVID-19 (Quick et al., 2016; Quick et al., 2017). Theoretically, with the advantage of long-read lengths in nanopore sequencing, excessive sequencing reads for bacterial- or viral-haplotype assembly might not be required. Our results also showed that the analysis time of NanoCoV19 was controlled within 1 h from input of the 1,000 sequencing reads to end of analysis. Some studies showed that the whole processing time based on nanopore sequencing platforms such as ONT or Qitan Technology (QT) to detect SARS-CoV-2 and other respiratory viruses simultaneously within 6–10 h (Wang et al., 2021a). And the main time consumption was in the wet experimental and libraries sequencing steps. Thereby, we are trying and foresee that the combination of real-time analysis in NST with more-advanced computing resources could control overall analysis time from sample collection to analysis report issuance to within 30 min or even less, yielding significant social and economic benefits. Although ONT’s sequencing solutions for SARS-CoV-2 have been established and applied in public-health scenarios (Meredith et al., 2020; Paden et al., 2020), the adoption of this technology has been somewhat limited due to concerns over sequencing accuracy. Given the technical principles and data characteristics of NST (Magi et al., 2017), such as non-random systematic errors and many unexpected indels, the accuracy of SARS-CoV-2 analysis results might be seriously affected. For example, we know that viruses are characterized by low mutation rates (Rambaut, 2020), so sequencing errors might lead to false-positive or false-negative assay results. Therefore, multi-dimensional or multi-aspect consideration, combination, and optimizing iteration may be required for analysis, especially for the infectious virus like SARS-CoV-2.

Although NanoCoV19 benefits in effectiveness from the combination of phylogenetic-tree and hotspot mutation analysis, it still has some shortcomings: 1) The accuracy and sufficiency of the constructed reference sequences and hotspot mutations database in viral-lineage discrimination still need further validation. 2) Continued optimization of the assembly method is still necessary due to the varying performances of different assembly algorithms for assembly results with the same data. For example, we also tried to conduct an assembly analysis on the simulated data using Raven and obtained results that were basically similar to those of FlyE, while the compositions of the outlier samples were different. This confirmed the necessity and high requirements for the quality and integrity of the assembly results before phylogenetic-tree analysis. Therefore, we used Trycycler to integrate multiple assemblies and generate consensus sequence, which is also a more important and worthy of attention in the NanoCoV19 analytical pipeline. However, the intermediate steps required manual selection of the better assembly results so that automation was insufficient. For example, the length of the assembly draft genome and/or the number of scaffolds were very different, so it was necessary to select or even delete some assemblies. Therefore, a method similar to MAECI (Lang, 2022) might also be required to balance accuracy and automation in the assembly results. 3) More tools and/or algorithms are needed for hotspot mutation detection [e.g., PEPPER-Margin-DeepVariant (Shafin et al., 2021) and Nano2NGS-Muta (Lang et al., 2022b)]. 4) NanoCoV19 should be further optimized for analysis time. Some steps could be run in parallel to shorten overall analysis time, although excessive memory consumption might happen, which would require a trade-off between resource consumption and analysis time. 5) As we known, SARS-CoV-2 virus strains are constantly evolving, resulting in the possible generation of many new strain genomes, so the relevant database will be continuously updated. However, NanoCoV19 only analyzes viral lineages with constructed reference database. Knowledge of determination criteria and processing methods for novel (unclassified) lineages is lacking. Therefore, a timely update of the reference database for the complete genome sequences is also required. 6) More actual data validation of NanoCoV19 performance is needed because the published raw sequencing data of SARS-CoV-2 genomes based on nanopore sequencing data are limited.

In summary, we hope that NanoCoV19 can be used as an auxiliary tool for rapid detection and lineage analysis of SARS-CoV-2, and that nanopore sequencers’ outstanding advantages of long-read length and real-time sequencing can provide faster and more-accurate solutions for genomic epidemiological surveillance. This would promote the application of NST in the fields of public-health planning and safety, and even offline applications in the international space stations (Castro-Wallace et al., 2017; Carr et al., 2020; Stahl-Rommel et al., 2021).

## 5 Conclusion

NanoCoV19 is a potential auxiliary tool for rapid detection and lineage analysis of SARS-CoV-2 based on nanopore sequencing technology. It completes all analysis within 1 h. We hope that it not only can assist in current-day lineage analysis and monitoring of SARS-CoV-2 but also promote the application of NST in related scientific research and clinical settings.

## Data Availability

The link to the RCoV19 database is https://ngdc.cncb.ac.cn/ncov/?lang=en. The SARS-CoV-2’s information is also downloaded from the SARS-CoV-2 Data Hub in the National Center for Biotechnology Information (NCBI; Bethesda, MD, USA) virus database. The codes are available at https://github.com/langjidong/NanoCoV19.
